# Surgical and Oncologic Outcomes of Tumescence and Sharp Dissection Versus Electrocautery Dissection in Minimal-Access Nipple-Sparing Mastectomy with Immediate Prosthesis Breast Reconstruction: A Real-World Retrospective Cohort Study

**DOI:** 10.1245/s10434-025-17680-4

**Published:** 2025-06-25

**Authors:** Xinyu Ou, Zhihan Liu, Caigang Liu, Kun Wang, Pusheng Zhang, Xuli Meng, Wei Wei, Yuan Shi, Shu Liu, Taolang Li, Tai Xu, Wei Tang, Chenlu Liu, Jiangtao Li, Zixuan Li, Jianli Zhao, Yan Nie, GuangLei Chen, Zhenye Lv, Xiaoling Liu, Yandan Yao, Yiwen Lu, Shicheng Su

**Affiliations:** 1 Breast Tumor Center, Sun Yat-Sen Memorial Hospital, Sun Yat-Sen University, Guangzhou, China; 2 Guangdong Provincial Key Laboratory of Malignant Tumor Epigenetics and Gene Regulation, Medical Research Center, Sun Yat-Sen Memorial Hospital, Sun Yat-Sen University, Guangzhou, China; 3 Department of Oncology, Shengjing Hospital of China Medical University, Shenyang, China; 4 Department of Breast Oncology, Guangdong Provincial People’s Hospital, Guangzhou, China; 5 Department of Breast Surgery, Zhujiang Hospital, Southern Medical University, Guangzhou, China; 6 General Surgery, Cancer Center, Department of Breast Surgery, Zhejiang Provincial People’s Hospital of Hangzhou Medical College, Hangzhou, China; 7 Department of Breast and Thyroid Surgery, Peking University Shenzhen Hospital, Shenzhen, China; 8 Department of Breast Surgery, Anyang Tumor Hospital, Anyang, China; 9 Department of Breast Surgery, The Affiliated Hospital of Guizhou Medical University, Guizhou, China; 10 Department of General Surgery, The Affiliated Hospital of Zunyi Medical University, Zunyi, Guizhou, China; 11 Department of Breast Surgery, Meizhou People’s Hospital, Meizhou, China; 12 Department of Breast Surgery, The First Affiliated Hospital, Guangzhou Medical University, Guangzhou, China; 13 Department of Infectious Diseases, Third Affiliated Hospital, Sun Yat-Sen University, Guangzhou, China; 14 Department of Immunology and Microbiology, Zhongshan School of Medicine, Sun Yat-Sen University, Guangzhou, China; 15 Biotherapy Center, Sun Yat-Sen Memorial Hospital, Sun Yat-Sen University, Guangzhou, China

**Keywords:** Nipple-sparing mastectomy, Minimal-access breast surgery, Immediate prosthesis breast reconstruction, Skin flap development, Surgical outcomes

## Abstract

**Background:**

Minimal-access nipple-sparing mastectomy (NSM) with immediate prosthesis breast reconstruction results in better cosmetic outcomes than conventional operation. However, the impact of skin flap development techniques on postoperative complications and long-term oncologic safety is largely unknown. This report describes the surgical and oncologic outcomes of tumescence and sharp dissection compared with electrocautery dissection.

**Methods:**

In this real-world retrospective cohort study, 5436 individuals undergoing NSM from 12 centers in China were identified. After exclusions and propensity score-matching, the study included 1252 patients who underwent minimal-access NSM with immediate prosthesis breast reconstruction between January 2016 and December 2022. Perioperative parameters, postsurgical complications, and long-term survival were analyzed.

**Results:**

Of the 1252 patients, 313 (25 %) underwent tumescence and sharp dissection, and 939 (75 %) underwent electrocautery dissection. The patients in the tumescence and sharp dissection group had significantly lower rates of necrotic complications (5.8 % vs 13.0 %; *p* = 0.001), infection (2.6 % vs 5.6 %; *p* = 0.041), and implant loss (0.3 % vs 2.2 %; *p* = 0.025) than those receiving electrocautery dissection, with a significantly shorter operation time (median, 177 min; interquartile range [IQR], 132–219 min vs 201 min; IQR, 143–249 min; *p *< 0.001). The two groups did not differ significantly in 5-year overall survival (*p* = 0.938) or disease-free survival (*p* = 0.893).

**Conclusion:**

Tumescence and sharp dissection was associated with fewer postoperative complications and a shorter operation time than electrocautery dissection for breast cancer patients receiving minimal-access NSM with immediate prosthesis breast reconstruction, and showed no significant difference in long-term survival.

**Supplementary Information:**

The online version contains supplementary material available at 10.1245/s10434-025-17680-4.

Because of superior aesthetic outcomes and equivalent oncologic outcomes, the use of minimal-access breast surgery has rapidly increased in recent years.^1,2^ The conventional open surgical technique is limited by conspicuous incision on the breast and compromised exposure of the superior pole with access from the inframammary fold.^3–5^

Minimal-access breast surgery is characterized by enhanced visualization through endoscopic or robotic cameras, hidden incision in the axilla, and improved preservation of the breast envelope.^1^ However, minimal-access breast surgery is yet to be widely adopted because of its drawbacks, such as a longer operation time and limited working space.^6–9^

About half of patients with breast cancer receive mastectomy.^10,11^ Nipple-sparing mastectomy (NSM) with immediate prosthesis breast reconstruction provides a good cosmetic effect by conserving skin and the nipple-areola complex and preserving a general symmetry with the contralateral breast.^12–14^ Open NSM generates a conspicuous scar.^15^ By comparison, minimal-access NSM, including endoscopic- and robotic-assisted NSM, conceals the incision in the axilla and therefore achieves better aesthetic outcomes and greater patient satisfaction, with a long-term safety.^1,2,6–9,16^

Necrotic complication, which includes nipple-areola complex necrosis and skin flap necrosis, is one of the most common complications of NSM, with incident rates ranging from about 13 % to 30 %.^17–23^ It extends the hospital length of stay, reduces cosmetic outcome, and increases the rate of infection. It is especially concerning in an implant-based reconstruction because it may lead to implant extrusion and reoperation.

As a key factor linked to flap blood supply, the dissection technique for skin flap development is of specific interest with regard to the risk of postoperative necrotic complications. Monopolar electrocautery has been shown to cause peripheral tissue temperature elevation and may impair the blood supply of the nipple-areola complex and skin flap.^24^

It has been well documented that using sharp dissection with scissors or scalpels after tumescence injection, compared with electrocautery, is related to lower rates of necrotic complications in open mastectomy.^20,25–27^ Skin flap development is even more challenging in minimal-access breast surgery because the working space is small and flap thickness can hardly be assessed by hand during this procedure.

Oncologic safety is paramount in the evaluation of surgical innovations for breast cancer. Previous studies have reported equivalent oncologic outcomes between minimal-access breast surgery and conventional breast surgery.^1,28^ However, comparative study investigating the oncologic outcomes of different dissection techniques in minimal-access breast surgery still is lacking.

In this study, we performed a real-world large-scale multicenter retrospective analysis to evaluate the surgical and oncologic outcomes of tumescence and sharp dissection compared with electrocautery dissection in breast cancer patients who received minimal-access NSM with immediate prosthesis breast reconstruction.

## Methods

### Study Designs and Population

For inclusion, the study evaluated 5436 patients with breast cancer from 12 hospitals in China who underwent NSM between January 2016 and December 2022. These 12 hospitals were Sun Yat-Sen Memorial Hospital, Shengjing Hospital of China Medical University, Guangdong Provincial People's Hospital, Zhujiang Hospital of Southern Medical University, Zhejiang Provincial People’s Hospital, Peking University Shenzhen Hospital, Anyang Tumor Hospital, the Affiliated Hospital of Guizhou Medical University, the Affiliated Hospital of Zunyi Medical University, Meizhou People's Hospital, the First Affiliated Hospital of Guangzhou Medical University, and Shenshan Center of Sun Yat-Sen Memorial Hospital.

Before study enrollment, we surveyed medical records from each center and collected the number of minimal-access NSMs with immediate prosthesis breast reconstruction cases accomplished by each surgeon. All the surgeons participating this study had a cumulative experience of more than 20 minimal-access NSM cases with immediate prosthesis breast reconstruction before performing the operation on the patients included in this study.

Data on patients’ demographics, perioperative parameters, postoperative complications, oncologic outcomes, and other related information were collected. We excluded patients with incomplete basic information (*n* = 306), missing follow-up data (*n* = 179), bilateral surgery (*n* = 95), or open surgery or without immediate prosthesis breast reconstruction (*n* = 3387), and those younger than 18 years as well as those older than 75 years (*n* =43). A total of 1426 individuals were assessed for inclusion.

This study was approved by the Sun Yat-sen Memorial Hospital review board (institutional review board no. SKSKY-2022-371-03) on 11 August 2023, and the requirement for informed consent was waived due to the retrospective nature of the study. All the hospitals obtained their respective approvals according to their local center’s requirements.

### Propensity Score-Matching

Propensity score-matching (PSM) was used to balance important baseline characteristics and reduce potential selection bias.^29,30^ We adhered to the following rules in choosing the covariates for PSM: (1) potential baseline differences between groups with a *p* value lower than 0.05 and (2) potentially relevant variables according to previous studies and clinical considerations. As a result, age, body mass index (BMI), smoking status, diabetes, and hormone receptor status were involved. The propensity score was calculated by logistic regression analysis using the R software MatchIt package (R Foundation for Statistical Computing, Vienna, Austria) and 1:3 nearest-neighbor matching.^31^ After PSM, the tumescence and sharp dissection group included 313 patients, and the electrocautery dissection group included 939 patients.

### Surgical Techniques

Minimal-access NSM with immediate prosthesis breast reconstruction in this study was performed as previously described.^2,32–34^ The standard anterior axillary incision of 3 to 8 cm was made for axillary lymph node biopsy/dissection, dissection of mammary glanduar tissue, and breast reconstruction. In the electrocautery dissection group, skin flap development was performed using monopolar cautery or bipolar cautery. Tumescent solution injection was not applied for the patients in the electrocautery dissection group.

In the tumescence and sharp dissection group, tumescent solution was injected into the subcutaneous tissue before skin flap development, and scalpels or scissors were used for skin flap development. The tumescent solution was saline containing 0.05 % lidocaine and 1:1,000,000 epinephrine.^35^

### Definitions and Data Collection

Perioperative parameters, postsurgical complications, and oncologic outcomes up to 7 years were obtained from the database of the 12 hospitals and carefully reviewed. The perioperative parameters were duration of the operation, estimated blood loss, drain volume, and length of hospital stay. Postoperative complications such as hematoma, seroma, necrotic complications including nipple-areola complex necrosis and skin flap necrosis, infection with implant loss, infection without implant loss, and capsular contraction were compared. Necrosis included both partial-thickness necrosis and full-thickness necrosis. Partial-thickness necrosis was defined as any minor superficial epidermolysis or delayed wound-healing in the nipple-areola complex or skin flap within 1 month after surgery. Full‐thickness necrosis was defined as any extrusion of acellular dermal matrix, muscle, or implant in the nipple-areola complex or skin flap within 1 month after surgery.^20,36^ The definition of infection was based on the Centers for Disease Control and Prevention criteria as follows: presence of purulent drainage, positive aseptically obtained culture result, peri-incisional erythema and incision opened by the surgeon, or physician diagnosis of infection, such as cellulitis, for which antibiotics were prescribed.^37^ Hematoma was defined as discoloration of the skin (red, purple, green, or otherwise) and a palpable lump in the surgically treated breast.^38^ Seroma was defined as persistent seroma requiring multiple aspirations for more than 6 weeks after removal of drains.^2^ The drain volume in our study was defined as the total amount of drainage during the hospital stay within 3 days after surgery. The oncologic outcomes included 5-year overall survival (OS) and disease-free survival (DFS). The follow-up period started on the date of surgery and ended on February 2024.

### Statistical Analysis

Continuous data are reported as medians with interquartile ranges (IQRs). Categorical data are reported as counts and percentages. Comparisons between the two groups were performed using the Mann‐Whitney *U* test for continuous variables and the chi-square test or Fisher’s exact test for categorical variables.

Both DFS and OS were evaluated with Kaplan-Meier survival curves analysis. The log-rank test was used for between-group comparisons. The median follow-up time was estimated by the reverse Kaplan-Meier method. Cox proportional hazards models were used to estimate hazard ratios (HRs) and 95 % confidence intervals (CIs).

Uni- and multivariable logistic regressions were used to estimate odds ratios (ORs) with 95 % CIs for necrotic complications. To reduce selection bias, stratified analyses were performed for age (categorized as ≤50, 51–60, or 61–75 years), BMI (categorized as <24, 24–27.9, or ≥28 kg/m^2^ based on the Chinese BMI criteria), and smoking status (categorized as never smoker, former smoker, or current smoker) (Table S1).^39–42^ Current smoker referred to a patient who smoked cigarettes daily or on some days at the time of the enrollment survey. Former smoker referred to a patient who had smoked more than 100 cigarettes in her lifetime but had quit smoking before surgery. Never smoker referred to an adult who consumed fewer than 100 cigarettes in her lifetime.^43^

Moreover, to further validate our findings, we first performed crude analyses adjusted only for age and only for current smoker, which may influence risks of necrotic complications.^40^ Then we additionally included the following covariates in the final fully adjusted model: age, BMI, current smoker, diabetes, tumor size, type of cancer, hormone receptor status, human epidermal growth factor receptor 2 (ERBB2 or HER2) status, preoperative chemotherapy, postoperative chemotherapy, estimated blood loss, and duration of operation, which may be potentially associated with risks of necrotic complications.

Statistical model information is provided in Table S2. All tests were two-sided, and a *p* value lower than 0.05 was considered significant. All statistics were computed using R version 4.3.0 (R Foundation) and SPSS version 25 (IBM Corp., Armonk, NY, USA).

## Results

**Fig. 1:**
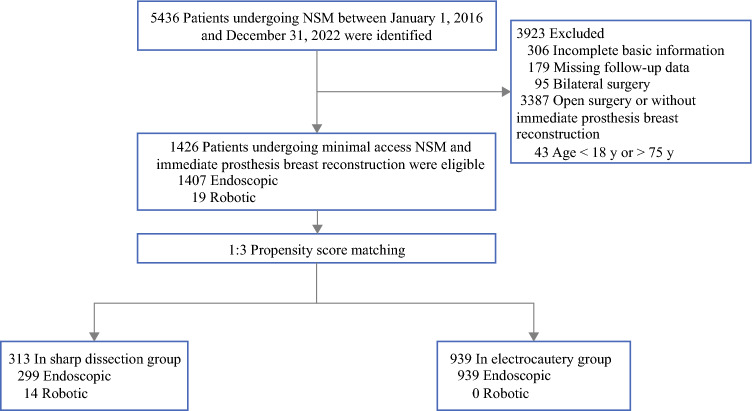
Flow diagram of participant selection. NSM, nipple-sparing mastectomy

For this study, 1426 female breast cancer patients from 12 hospitals in China who underwent minimal-access NSM and immediate prosthesis breast reconstruction, performed by 15 experienced surgeons, were eligible (Fig. 1). Among these patients, 313 received tumescence and sharp skin flap development, and 1113 received electrocautery skin flap development. After PSM, 313 were included in the tumescence and sharp dissection group, and 939 patients were included in the electrocautery dissection group. The tumescence and sharp dissection group included 14 cases operated with robotic-assisted techniques, and no case received robot-assisted surgery in the electrocautery dissection cohort.

### Baseline Characteristics

The demographic and clinical characteristics of the study population before and after PSM are shown in Table 1. After PSM, the two groups did not differ significantly in age, BMI, smoking status, diabetes, tumor size, lymph node status, type of cancer, hormone receptor status, ERBB2 status, preoperative chemotherapy, postoperative chemotherapy, or lymph node surgery.

**Table 1:** Baseline characteristics of patients receiving minimal-access NSM with immediate prosthesis breast reconstruction using tumescence and sharp dissection versus electrocautery dissection before and after propensity score-matching

Variable	Before PSM No. (%)			After PSM No. (%)		
	Tumescence and sharp dissection (*n* = 313) *n* (%)	Electrocautery dissection (*n* = 1113) *n* (%)	*P* value	Tumescence and sharp dissection (*n* = 313) *n* (%)	Electrocautery dissection (*n* = 939) *n* (%)	*p* value
Age (years)			0.163			0.225
≤50	241 (77.0)	899 (80.8)		241 (77.0)	755 (80.4)	
>50	72 (23.0)	214 (19.2)		72 (23.0)	184 (19.6)	
BMI (kg/m^2^)			0.349			0.460
<28	290 (92.7)	1010 (90.7)		290 (92.7)	883 (94.0)	
≥28	23 (7.3)	103 (9.3)		23 (7.3)	56 (6.0)	
Smoking status			0.032^a^			0.080
Never smoker	297 (94.9)	1087 (97.7)		297 (94.9)	915 (97.4)	
Former smoker	6 (1.9)	8 (0.7)		6 (1.9)	8 (0.9)	
Current smoker	10 (3.2)	18 (1.6)		10 (3.2)	16 (1.7)	
Diabetes			0.038^a^			0.128
No	307 (98.1)	1060 (95.2)		307 (98.1)	902 (96.1)	
Yes	6 (1.9)	53 (4.8)		6 (1.9)	37 (3.9)	
Tumor size (cm)			0.758			>0.99
≤2.0	98 (31.3)	361 (32.4)		98 (31.3)	292 (31.1)	
2.1–5.0	215 (68.7)	752 (67.6)		215 (68.7)	647 (68.9)	
Lymph node status			0.659			0.326
Negative	151 (48.2)	519 (46.6)		151 (48.2)	421 (44.8)	
Positive	162 (51.8)	594 (53.4)		162 (51.8)	518 (55.2)	
Type of cancer			0.993			0.620
*In situ*	26 (8.3)	90 (8.1)		26 (8.3)	68 (7.2)	
Invasive	287 (91.7)	1023 (91.9)		287 (91.7)	871 (92.8)	
Hormone receptor status			0.005^a^			0.093
Negative	35 (11.2)	201 (18.1)		35 (11.2)	143 (15.2)	
Positive	278 (88.8)	912 (81.9)		278 (88.8)	796 (84.8)	
ERBB2 status			0.307			0.402
Negative	220 (70.3)	817 (73.4)		220 (70.3)	685 (72.9)	
Positive	93 (29.7)	296 (26.6)		93 (29.7)	254 (27.1)	
Preoperative chemotherapy			0.711			0.180
No	177 (56.5)	614 (55.2)		177 (56.5)	488 (52.0)	
Yes	136 (43.5)	499 (44.8)		136 (43.5)	451 (48.0)	
Postoperative chemotherapy			0.245			0.645
No	55 (17.6)	231 (20.8)		55 (17.6)	178 (19.0)	
Yes	258 (82.4)	882 (79.2)		258 (82.4)	761 (81.0)	
Lymph node surgery			0.817			0.593
SLNB only	127 (40.6)	462 (41.5)		127 (40.6)	363 (38.7)	
ALND	186 (59.4)	651 (58.5)		186 (59.4)	576 (61.3)	

*NSM* nipple-sparing mastectomy, *PSM* propensity score-matching, *BMI* body mass index, *SLNB* sentinel lymph node biopsy, *ALND* axillary lymph node dissection

^a ^indicates statistical significance (*p* < 0.050)

### Perioperative Parameters

The patients in the tumescence and sharp dissection group had a significantly shorter operation (median, 177 min; interquartile range [IQR], 132–219 min) than those in the electrocautery dissection group (median, 201 min; IQR, 143–249 min) (*p <* 0.001) (Table 2). The postoperative drainage volume was greater for the patients in the tumescence and sharp dissection group (median, 342 ml; IQR, 296–408 ml) than for those in the electrocautery dissection group (median, 261 ml; IQR, 165– 343.0 ml) (*p*< 0.001). No statistical difference was observed between the patients in the tumescence and sharp dissection group and the electrocautery dissection group in terms of blood loss (median, 40 ml [IQR, 20–55 ml] vs median, 40 ml; IQR, 20–50 ml; *p* = 0.891) or hospital length of stay (median 7 days [IQR, 5–10 days] vs 7 days [IQR, 5–9 days]; *p* = 0.459) (Table 2).

**Table 2:** Perioperative parameters of patients receiving the tumescence and sharp dissection versus electrocautery dissection during minimal-access NSM with immediate prosthesis breast reconstruction

Parameter	Tumescence and sharp dissection (*n* = 313) *Median* (IQR)	Electrocautery dissection (*n* = 939) *Median* (IQR)	*p* Value
Median duration of operation (min)	177.0 (132.0–219.0)	201.0 (143.0–249.0)	<0.001^a^
Median EBL (ml)	40.0 (20.0–55.0)	40.0 (20.0–50.0)	0.891
Median drain volume (ml)	342.0 (296.0–408.0)	261.0 (165.0–343.0)	<0.001^a^
Median hospital stay (days)	7.0 (5.0–10.0)	7.0 (5.0– 9.0)	0.459

*NSM* nipple-sparing mastectomy, *IQR* interquartile range, *EBL* estimated blood loss

^a^indicates statistical significance (*p* < 0.050)

### Postoperative Complications

The rates of postoperative necrotic complications, including skin flap necrosis alone (1.9 % [6 of 313 patients] vs 5.2 % [49 of 939 patients; *p* = 0.021) and nipple-areola complex necrosis alone (3.2 % [10 of 313 patients] vs 6.6 % [62 of 939 patients]; *p* = 0.036), were significantly lower in the tumescence and sharp dissection group (5.8 % [18 of 313 patients]) than in the electrocautery dissection group (13.0 % [122 of 939 patients] (*p* = 0.001; Table 3). Furthermore, fewer patients experienced infection (8 patients [2.6 %] vs 53 patients [5.6 %]; *p* = 0.041) or implant loss (1 patients [0.3 %] vs 21 patients [2.2 %]; *p* = 0.025) in the tumescence and sharp dissection group than in the electrocautery dissection group. No significant difference was observed in other postoperative complications (Table 3).

**Table 3:** The rate of postoperative complications in patients receiving tumescence and sharp dissection versus electrocautery dissection during minimal-access NSM with immediate prosthesis breast reconstruction

Outcome	Tumescence and sharp dissection (*n* = 313) *n* (%)	Electrocautery dissection (*n* = 939) *n* (%)	*p* Value
Hematoma	3 (1.0)	9 (1.0)	>0.99
Seroma	7 (2.2)	30 (3.2)	0.500
Necrotic complications	18 (5.8)	122 (13.0)	0.001^a^
Skin flap necrosis alone	6 (1.9)	49 (5.2)	0.021^a^
NAC necrosis alone	10 (3.2)	62 (6.6)	0.036^a^
NAC necrosis with skin flap necrosis	2 (0.6)	11 (1.2)	0.629
Infection	8 (2.6)	53 (5.6)	0.041^a^
Infection with implant loss	1 (0.3)	21 (2.2)	0.025^a^
Infection without implant loss	7 (2.2)	32 (3.4)	0.398
Capsular contraction	7 (2.2)	28 (3.0)	0.621

*NSM* nipple-sparing mastectomy, *NAC* nipple-areola complex

^a^indicates statistical significance (*p* < 0.050)

### Uni- and Multivariable Analyses

In the univariate analyses, the higher rate of necrotic complications was associated with age older than 50 years (OR, 1.66; 95 % CI, 1.12–2.46; *p* = 0.012) and current smoker status (OR, 2.77; 95 % CI, 1.33–5.81, *p* = 0.007) (Table 4). Moreover, tumescence and sharp skin flap development was correlated with a lower risk of necrotic complications (OR, 0.41; 95 % CI, 0.24–0.68; *p* < 0.001). The association between necrotic complications and age older than 50 years (OR, 1.53; 95 % CI, 1.00–2.35; *p* = 0.049), current smoker status (OR, 2.65; 95 % CI, 1.18–5.95; *p* = 0.018), and tumescence and sharp skin flap development (OR, 0.40; 95 % CI, 0.24–0.67; *p* < 0.001) persisted in multivariate analyses (Table 4).

**Table 4:** Uni- and multivariable analyses for risks of necrotic complications of patients receiving minimal-access NSM with immediate prosthesis breast reconstruction using tumescence and sharp dissection versus electrocautery dissection after propensity score- matching

	Univariate		Multivariable	
Parameter	OR (95 % CI)	*p* value	OR (95 % CI)	*p* value
Age >50 years	1.66 (1.12–2.46)	0.012^a^	1.53 (1.00–2.35)	0.049^a^
BMI ≥28 kg/m^2^	1.16 (0.58–2.31)	0.667	–	–
Diabetes, yes	1.30 (0.54–3.14)	0.558	–	–
Current smoker, yes	2.77 (1.33–5.81)	0.007^a^	2.65 (1.18–5.95)	0.018^a^
Duration of operation >196 min	1.35 (0.95–1.92)	0.099	–	–
Estimated blood loss, >40 ml	0.86 (0.60–1.22)	0.393	–	–
Tumescence and sharp skin flap development, yes	0.41 (0.24–0.68)	<0.001^a^	0.40 (0.24–0.67)	<0.001^a^

*NSM* nipple-sparing mastectomy, *OR* odds ratio, *CI* confidence interval, *BMI* body mass index

^a^indicates statistical significance (*p* < 0.050)

### Oncologic Outcomes

**Fig. 2:**
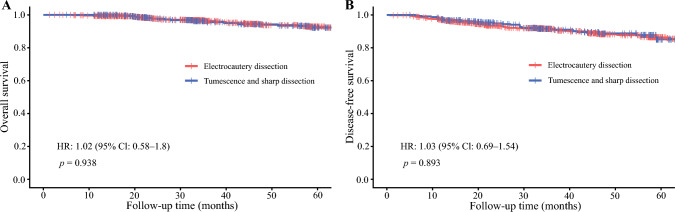
Kaplan-Meier survival curve after propensity score-matching. Kaplan-Meier survival curves showing the 5-year **A** overall survival and **B** disease-free survival of breast cancer patients receiving minimal-access nipple-sparing mastectomy with immediate prosthesis breast reconstruction using tumescence and sharp dissection versus electrocautery dissection after propensity score-matching

The median follow-up time after PSM was 38.3 months (IQR, 35.5–44.4 months) in the electrocautery dissection group and 35.9 months (IQR, 34.4–44.8 months) in the tumescence and sharp dissection group (*p* = 0.336). The 5-year OS rate was 92.2 % in the electrocautery dissection group and 92.8 % in the tumescence and sharp dissection group. The 5-year DFS rate was 85.1 % in the electrocautery dissection group and 86.4 % in the tumescence and sharp dissection group. No significant difference was observed between the two groups in 5-year OS (HR, 1.02; 95 % CI, 0.58–1.80; *p* = 0.938) or DFS (HR, 1.03; 95 % CI, 0.69–1.54; *p* =0.893) (Fig. 2A and B).

## Discussion

The trends of endoscopic- and robotic-assisted breast surgery have continued to increase rapidly over the decades.^1^ Among these, minimal-access NSM with immediate prosthesis breast reconstruction is one of the most frequently performed procedures. However, skin flap necrosis is a major problem after NSM.

The comparative risks of surgical complications with tumescence and sharp dissection versus electrocautery dissection in conventional open mastectomy have been extensively studied, although controversial findings exist. A retrospective study demonstrated that the tumescent technique was associated with a reduced risk of buttonholing of the skin, elimination of burn artifact potential, and maintenance of flap uniformity compared with electrocautery techniques.^44^ Another study showed a similar result and reported that the tumescent technique may reduce skin flap necrosis by avoiding thermal energy dissipation and obviating pressure on the flap, which may impact blood flow during surgery.^20^

In contrast, studies have shown an association of tumescence and sharp dissection with a higher risk of flap necrosis due to the epinephrine-induced vasoconstriction.^40,45^ It also has been reported that reduced blood flow may result in decreased oxygen and potential tissue damage.^46^ In addition, some studies have found no statistically significant difference in skin necrosis rates between patients receiving tumescence and sharp dissection and those receiving electrocautery dissection.^47–49^ A prior study showed that the risks of postoperative complications, such as infection and seroma, differ significantly between open and minimal-access mastectomy.^50^

Although the association between postoperative complications and dissection techniques in open mastectomy has been well-characterized, the impact of dissection equipment on surgical outcomes in minimal-access surgery has not been evaluated to date. Moreover, whether the dissection techniques are associated with oncologic outcomes in mastectomy is also unknown.

For the first time, our study showed that tumescence and sharp dissection was associated with fewer postoperative complications and a shorter operation time while maintaining a comparable long-term survival compared with electrocautery dissection in minimal-access NSM with immediate prosthesis breast reconstruction. In addition, it was a large-scale multicenter study with detailed perioperative, postoperative, and oncologic follow-up data.

To ensure comparability between cohorts and reduce selection bias, PSM was performed. All procedures were performed by surgeons experienced in minimal-access NSM with immediate prosthesis breast reconstruction. We report that the patients who underwent minimal-access NSM with immediate prosthesis breast reconstruction using tumescence and sharp skin flap development had lower risks of necrotic complications than those with electrocautery skin flap development (5.8 % vs 13.0 %; *p* = 0.001).

Currently, direct evidence for technique selection in endoscopic/robotic NSM still is lacking. Although the latest consensus statement on robotic mastectomy recommends meticulous dissection using scissors without the use of coagulation after tumescent injection for skin flap development, direct evidence still is lacking.^51^ Moreover, the risk factor for skin flap necrosis in minimal-access NSM remains unclear. Our study provided direct evidence for the use of tumescence and sharp dissection in minimal-access NSM and suggests age and current smoker status as risk factors for necrotic complications after minimal-access NSM with immediate prosthesis breast reconstruction.

Preventing skin flap necrosis is more important for patients with immediate prosthesis breast reconstruction than for those receiving no reconstruction or autologous reconstruction. It has been reported that breast cancer patients receiving immediate prosthesis breast reconstruction have a higher rate of postoperative complications than those receiving autologous reconstruction.^15^ Skin flap necrosis impairs the skin barrier and provides access for microbe invasion.^52,53^ What is worse, the presence of an artificial implant fosters a proinflammatory response, which may lead to prolonged infection, prothesis loss, reoperation, increased medical expenses and even life-threating sepsis.^52–54^ Consistently, our data demonstrated that the infection rate was significantly lower for the tumescence and sharp dissection group together with the reduced incidence of skin flap necrosis.

Another interesting finding was the significant association of tumescence and sharp skin flap development with a shorter operation time. The concern of skin blood supply damage and skin perforation by electrocautery probably makes surgeons more prudent during skin flap development and therefore reduces their operating speed. A concern about sharp skin flap development by scalpels or scissors is hemostasis. The perfusion of tumescence solution creates a bloodless plane. It has been reported that tumescence separation even results in less blood loss than non-tumescence separation in open breast reduction surgery.^55^ In our study, although postoperative drainage volume was higher in the tumescence and sharp dissection group, neither the estimated blood loss nor the rates of postoperative hematoma and seroma differed significantly between the two groups.

Previous studies have identified older age, obesity, diabetes, and current smoker status as potential risk factors for nipple-areola complex or skin flap necrosis.^36,54,56,57^ A number of baseline and surgical factors and their association with necrotic complications were reviewed in our analysis. Our results demonstrated that older age and current smoker status were associated with an increased risk of necrotic complications in minimal-access NSM with immediate prosthesis breast reconstruction. However, we did not find a significant association between necrotic complications and a BMI of at least 28 kg/m^2^ or diabetes.

Surgical volume and expertise are important factors associated with clinical outcomes in a large-scale surgery-related multicenter study.^58^ Sufficient time is needed for skill mastery by surgeons.^59^

The first minimal-access NSM with immediate prosthesis breast reconstruction in China was performed in the 1990s.^60^ Surgical technique has evolved substantially since then, and the minimal-access technique has been extensively applied in breast surgery. In this study, we collected data from patients who received surgery from 2016 to 2024, when the technical skills of some surgeons had been proficient. In addition, previous retrospective studies accounted for skill mastery by including cases managed by highly experienced surgeons.^61,62^

In this study, all the included patients received surgeries performed by experienced surgeons who had a cumulative experience of more than 20 cases of minimal-access NSM with immediate prosthesis breast reconstruction. Besides, despite challenges in variation across sites, multicenter studies have many advantages including quicker recruitment, diverse population coverage, and increased generalizability. It has been reported that the multicenter nature of study design diminishes the effect of individual surgical performance and improves confidence in the generalizability of findings.^63,64^

Due to the retrospective nature of this study, several limitations merit discussion. First, although we collected comprehensive baseline characteristics, some variables such as anatomic factors (e.g., breast ptosis degree, cup size) that might influence surgical approach selection were not systematically recorded. This leads to potential selection bias.

Second, there were possible biases inherent in the multicenter nature of the study. Although all the surgeons adhered to standardized protocols, unmeasured variation in these morphologic characteristics could have introduced allocation bias. Although measurable confounders such as BMI and smoking status were balanced using PSM, biases may have persisted due to unmeasured variables.

Third, the median follow-up duration in this study was approximately 35 to 39 months, which is relatively short for oncologic safety assessment. Further prospective studies with extended follow-up time are needed to validate the comparable long-term oncologic safety of dissection techniques.

Finally, although all the surgeries of the included patients were performed by experienced surgeons in this study, generalizability is a ubiquitous concern in studies involving surgical experience and technique. Prospective clinical trials comparing surgical outcomes of these two procedures are warranted.

## Conclusions

Tumescence and sharp dissection was associated with fewer postoperative complications and a shorter operation time than electrocautery dissection in breast cancer patients receiving minimal-access NSM with immediate prosthesis breast reconstruction, with no significant difference in long-term survival. These findings may provide evidences for guideline recommendations.

## Supplementary Information

Below is the link to the electronic supplementary material.

Supplementary file1 (DOCX 19 kb)

## References

[1] Wan A, Liang Y, Chen L, et al. Association of long-term oncologic prognosis with minimal-access breast surgery vs conventional breast surgery. *JAMA Surg*. 2022;157:e224711. 36197680 10.1001/jamasurg.2022.4711 PMC9535498

[2] Lai HW, Chen DR, Liu LC, et al. Robotic versus conventional or endoscopic-assisted nipple-sparing mastectomy and immediate prosthesis breast reconstruction in the management of breast cancer: a prospectively designed multicenter trial comparing clinical outcomes, medical cost, and patient-reported outcomes (RCENSM-P). *Ann Surg*. 2024;279:138–46. 37226826 10.1097/SLA.0000000000005924 PMC10727200

[3] Lai H-W, Chen S-T, Lin Y-J, et al. Minimal-access (endoscopic and robotic) breast surgery in the surgical treatment of early breast cancer: trend and clinical outcome from a single-surgeon experience over 10 years. *Front Oncol.* 2021;11:739144. 34868935 10.3389/fonc.2021.739144 PMC8640170

[4] Leff DR, Vashisht R, Yongue G, et al. Endoscopic breast surgery: where are we now and what might the future hold for video-assisted breast surgery? *Breast Cancer Res Treat*. 2011;125:607–25. 21128113 10.1007/s10549-010-1258-4

[5] Mok CW, Lai H-W. Endoscopic-assisted surgery in the management of breast cancer: 20 years review of trend, techniques and outcomes. *Breast*. 2019;46:144–56. 31176887 10.1016/j.breast.2019.05.013

[6] Lai H-W, Chen S-T, Tai C-M, et al. Robotic- Versus endoscopic-assisted nipple-sparing mastectomy with immediate prosthesis breast reconstruction in the management of breast cancer: a case–control comparison study with analysis of clinical outcomes, learning curve, patient-reported aesthetic results, and medical cost. *Ann Surg Oncol*. 2020;27:2255–68. 32016631 10.1245/s10434-020-08223-0

[7] Margenthaler JA. Robotic mastectomy-program malfunction? *JAMA Surg*. 2020;155:461–2. 32236516 10.1001/jamasurg.2019.6361

[8] Yang H, Liang F, Xie Y, et al. Single axillary incision reverse-order endoscopic nipple/skin-sparing mastectomy followed by subpectoral implant-based breast reconstruction: technique, clinical outcomes, and aesthetic results from 88 preliminary procedures. *Surgery*. 2023;174:464–72. 37422354 10.1016/j.surg.2023.05.037

[9] Lai HW, Chen ST, Tai CM, et al. Robotic- versus endoscopic-assisted nipple-sparing mastectomy with immediate prosthesis breast reconstruction in the management of breast cancer: a case-control comparison study with analysis of clinical outcomes, learning curve, patient-reported aesthetic results, and medical cost. *Ann Surg Oncol*. 2020;27:2255–68. 32016631 10.1245/s10434-020-08223-0

[10] Galimberti V, Vicini E, Corso G, et al. Nipple-sparing and skin-sparing mastectomy: review of aims, oncological safety, and contraindications. *Breast*. 2017;34(Suppl 1):S82–4. 28673535 10.1016/j.breast.2017.06.034 PMC5837802

[11] Staradub VL, Morrow M. Modified radical mastectomy with knife technique. *Arch Surg*. 2002;137:105–10. 11772228 10.1001/archsurg.137.1.105

[12] Freeman BS. Subcutaneous mastectomy for benign breast lesions with immediate or delayed prosthetic replacement. *Plast Reconstr Surg Transplant Bull*. 1962;30:676–82. 13959443 10.1097/00006534-196212000-00008

[13] Santosa KB, Qi J, Kim HM, et al. Long-term patient-reported outcomes in postmastectomy breast reconstruction. *JAMA Surg*. 2018;153:891–9. 29926096 10.1001/jamasurg.2018.1677 PMC6233781

[14] Pusic AL, Matros E, Fine N, et al. Patient-reported outcomes 1 year after immediate breast reconstruction: results of the Mastectomy Reconstruction Outcomes Consortium Study. *J Clin Oncol *. 2017;35:2499–506. 28346808 10.1200/JCO.2016.69.9561 PMC5536162

[15] Bennett KG, Qi J, Kim HM, et al. Comparison of 2-year complication rates among common techniques for postmastectomy breast reconstruction. *JAMA Surg.* 2018;153:901–908. 29926077 10.1001/jamasurg.2018.1687 PMC6233788

[16] Toesca A, Sangalli C, Maisonneuve P, et al. A randomized trial of robotic mastectomy versus open surgery in women with breast cancer or BrCA mutation. *Ann Surg*. 2022;276:11–9. 34597010 10.1097/SLA.0000000000004969

[17] Yabe S, Nakagawa T, Oda G, et al. Association between skin flap necrosis and sarcopenia in patients who underwent total mastectomy. *Asian J Surg*. 2021;44:465–70. 33229126 10.1016/j.asjsur.2020.11.001

[18] Rinker B. A comparison of methods to assess mastectomy flap viability in skin-sparing mastectomy and immediate reconstruction: a prospective cohort study. *Plast Reconstr Surg*. 2016;137:395–401. 26818271 10.1097/01.prs.0000475744.10344.1e

[19] Phillips BT, Lanier ST, Conkling N, et al. Intraoperative perfusion techniques can accurately predict mastectomy skin flap necrosis in breast reconstruction: results of a prospective trial. *Plast Reconstr Surg*. 2012;129:778e-e788. 10.1097/PRS.0b013e31824a2ae8 22544108

[20] Ng T, Knowles S, Brackstone M, Doherty C. Mastectomy flap necrosis after nipple-sparing mastectomy and immediate implant-based reconstruction: an evaluation of tumescence and sharp dissection technique on surgical outcomes. *Breast J*. 2019;25:1079–83. 31359567 10.1111/tbj.13442

[21] Ryu JM, Kim JY, Choi HJ, et al. Robot-assisted nipple-sparing mastectomy with immediate breast reconstruction: an initial experience of the Korea Robot-Endoscopy Minimal-Access Breast Surgery Study Group (KoREa-BSG). *Ann Surg*. 2022;275:985–91. 32941285 10.1097/SLA.0000000000004492

[22] Jimenez RB, Packowski K, Horick N, et al. The timing of acute and late complications following mastectomy and implant-based reconstruction. *Ann Surg.* 2022;278: e203-8. 35837894 10.1097/SLA.0000000000005574

[23] Garwood ER, Moore D, Ewing C, et al. Total skin-sparing mastectomy: complications and local recurrence rates in 2 cohorts of patients. *Ann Surg*. 2009;249:26–32. 19106672 10.1097/SLA.0b013e31818e41a7

[24] Lantis JC II, Durville FM, Connolly R, et al. Comparison of coagulation modalities in surgery. *Laparoendosc Adv Surg Tech A*. 1998;8:381–94. 10.1089/lap.1998.8.381 9916591

[25] Gipponi M, Baldelli I, Atzori G, et al. Tumescent anesthesia in skin- and nipple-sparing mastectomy: results of a prospective clinical study. *Anticancer Res*. 2017;37:349–52. 28011513 10.21873/anticanres.11328

[26] Miller E, Paull DE, Morrissey K, et al. Scalpel versus electrocautery in modified radical mastectomy. *Am Surg*. 1988;54:284–6. 3364865

[27] Kurtz SB, Frost DB. A comparison of two surgical techniques for performing mastectomy. *EJSO Eur J Surg Oncol*. 1995;21:143–5. 7720886 10.1016/s0748-7983(95)90171-x

[28] Lai HW, Chen ST, Liao CY, et al. Oncologic outcome of endoscopic assisted breast surgery compared with conventional approach in breast cancer: an analysis of 3426 primary operable breast cancer patients from single institute with and without propensity score-matching. *Ann Surg Oncol*. 2021;28:7368–80. 33974198 10.1245/s10434-021-09950-8

[29] Austin PC. An introduction to propensity score methods for reducing the effects of confounding in observational studies. *Multivariate Behav Res*. 2011;46:399–424. 21818162 10.1080/00273171.2011.568786 PMC3144483

[30] Lobo FS, Wagner S, Gross CR, Schommer JC. Addressing the issue of channeling bias in observational studies with propensity scores analysis. *Res Social Adm Pharm*. 2006;2:143–51. 17138506 10.1016/j.sapharm.2005.12.001

[31] Ho D, Imai K, King G, Stuart EA. MatchIt: nonparametric preprocessing for parametric causal inference. *J Stat Software*. 2011;42:1–28.

[32] Lai HW, Lin SL, Chen ST, et al. Robotic nipple-sparing mastectomy and immediate breast reconstruction with gel implant. *Plast Reconstr Surg Glob Open*. 2018;6:e1828. 30276055 10.1097/GOX.0000000000001828 PMC6157943

[33] Hwang RF, Hunt KK. The emergence of robotic-assisted breast surgery: proceed with caution. *Ann Surg*. 2020;271:1013–5. 32398613 10.1097/SLA.0000000000003902

[34] Ryu JM, Lee J, Lee J, et al. Mastectomy with reconstruction including robotic endoscopic surgery (MARRES): a prospective cohort study of the Korea Robot-Endoscopy Minimal Access Breast Surgery Study Group (KoREa-BSG) and Korean Breast Cancer Study Group (KBCSG). *BMC Cancer*. 2023;23:571. 37344780 10.1186/s12885-023-10978-0 PMC10283322

[35] Chu P-Y, Lai H-W, Chen S-T, et al. Current trends in and indications for endoscopy-assisted breast surgery for breast cancer: results from a six-year study conducted by the Taiwan Endoscopic Breast Surgery Cooperative Group. *Plos One.* 2016;11:e0150310. 26950469 10.1371/journal.pone.0150310 PMC4780808

[36] Abedi N, Ho AL, Knox A, et al. Predictors of mastectomy flap necrosis in patients undergoing immediate breast reconstruction. *Ann Plast Surg*. 2016;76:629–34. 25003437 10.1097/SAP.0000000000000262

[37] Mangram AJ, Horan TC, Pearson ML, et al. Guideline for prevention of surgical-site infection, 1999. Centers for Disease Control and Prevention (CDC) Hospital Infection Control Practices Advisory Committee. *Am J Infect Control.* 1999;27:97–132; quiz 133–4; discussion 196. 10196487

[38] Somerville P, Seifert PJ, Destounis SV, et al. Anticoagulation and bleeding risk after core needle biopsy. *AJR Am J Roentgenol*. 2008;191:1194–7. 18806164 10.2214/AJR.07.3537

[39] Seth AK, Hirsch EM, Fine NA, et al. Additive risk of tumescent technique in patients undergoing mastectomy with immediate reconstruction. *Ann Surg Oncol*. 2011;18:3041–6. 21947584 10.1245/s10434-011-1913-y

[40] Chun YS, Verma K, Rosen H, et al. Use of tumescent mastectomy technique as a risk factor for native breast skin flap necrosis following immediate breast reconstruction. *Am J Surg*. 2011;201:160–5. 20409522 10.1016/j.amjsurg.2009.12.011

[41] Chen K, Shen Z, Gu W, et al. Prevalence of obesity and associated complications in China: a cross-sectional, real-world study in 15.8 million adults. *Diabetes Obes Metab.* 2023;25:3390–9. 10.1111/dom.15238 37589256

[42] Zhou BF. Predictive values of body mass index and waist circumference for risk factors of certain related diseases in Chinese adults: study on optimal cut-off points of body mass index and waist circumference in Chinese adults. *Biomed Environ Sci*. 2002;15:83–96. 12046553

[43] Cho ER, Brill IK, Gram IT, et al. Smoking cessation and short- and longer-term mortality. *NEJM Evid*. 2024;3:EVIDoa2300272. 10.1056/EVIDoa2300272 38329816

[44] Shoher A, Hekier R, Lucci A Jr. Mastectomy performed with scissors following tumescent solution injection. *J Surg Oncol*. 2003;83:191–3. 12827691 10.1002/jso.10265

[45] Barakat KE, Asal MF, Elsayed AAR, et al. Comparison between bipolar scissors, monopolar electrocautery, and hydrodissection in nipple-sparing mastectomy. *Surg Oncol*. 2025;58:102182. 39718307 10.1016/j.suronc.2024.102182

[46] Thalji SZ, Cortina CS, Guo MS, Kong AL. Postoperative complications from breast and axillary surgery. *Surg Clin North Am*. 2023;103:121–39. 36410345 10.1016/j.suc.2022.08.007

[47] Lautrup MD, Thomsen JB, Christensen RD, Kjaer C. Tumescent technique versus electrocautery mastectomy: a randomized controlled trial. *Surg Oncol*. 2020;34:276–82. 32891342 10.1016/j.suronc.2020.05.003

[48] Abbott AM, Miller BT, Tuttle TM. Outcomes after tumescence technique versus electrocautery mastectomy. *Ann Surg Oncol*. 2012;19:2607–11. 22402814 10.1245/s10434-012-2304-8

[49] Donovan CA, Harit AP, Chung A, et al. Oncological and surgical outcomes after nipple-sparing mastectomy: do incisions matter? *Ann Surg Oncol*. 2016;23:3226–31. 27352202 10.1245/s10434-016-5323-z

[50] Kim JH, Ryu JM, Bae SJ, et al. Minimal-access vs conventional nipple-sparing mastectomy. *JAMA Surg*. 2024;159:1177–86. 39141399 10.1001/jamasurg.2024.2977 PMC11325243

[51] Lai H-W, Toesca A, Sarfati B, et al. Consensus statement on robotic mastectomy: Expert panel from International Endoscopic and Robotic Breast Surgery Symposium (IERBS) 2019. *Ann Surg*. 2020;271:1005–12. 31977514 10.1097/SLA.0000000000003789

[52] Barr S, Hill EW, Bayat A. Functional biocompatibility testing of silicone breast implants and a novel classification system based on surface roughness. *J Mech Behav Biomed Mater*. 2017;75:75–81. 28697402 10.1016/j.jmbbm.2017.06.030

[53] Sarfati I, Millochau J, Meredith I, et al. Salvaging the infected breast implant: results of a retrospective series of 80 consecutive cases. *J Plast Reconstr Aesthet Surg*. 2020;73:2232–8. 32601014 10.1016/j.bjps.2020.05.042

[54] Al-Hilli Z, Wilkerson A. Breast surgery. *Surg Clin North Am*. 2021;101:845–63. 34537147 10.1016/j.suc.2021.06.014

[55] Uslu AB. Effect of tumescent lidocaine and epinephrine infiltration on blood loss in inferior pedicle wise-pattern breast reduction: a prospective randomized study. *Aesth Plast Surg*. 2020;45:442–50. 10.1007/s00266-020-01859-z 32671449

[56] Mlodinow AS, Fine NA, Khavanin N, Kim JY. Risk factors for mastectomy flap necrosis following immediate tissue expander breast reconstruction. *J Plast Surg Hand Surg*. 2014;48:322–6. 24495186 10.3109/2000656X.2014.884973

[57] Voineskos SH, Frank SG, Cordeiro PG. Breast reconstruction following conservative mastectomies: predictors of complications and outcomes. *Gland Surg*. 2015;4:484–96. 26645003 10.3978/j.issn.2227-684X.2015.04.13 PMC4647007

[58] Topal H, Aerts R, Laenen A, et al. Survival after minimally invasive vs open surgery for pancreatic adenocarcinoma. *JAMA Netw Open*. 2022;5:e2248147. 36547979 10.1001/jamanetworkopen.2022.48147 PMC9857028

[59] Tupper HI, Lawson BL, Kipnis P, et al. Video-assisted vs robotic-assisted lung lobectomies for operating room resource utilization and patient outcomes. *JAMA Netw Open*. 2024;7:e248881. 38700865 10.1001/jamanetworkopen.2024.8881 PMC11069083

[60] Ho WS, Ying SY, Chan AC. Endoscopic-assisted subcutaneous mastectomy and axillary dissection with immediate mammary prosthesis reconstruction for early breast cancer. *Surg Endosc*. 2002;16:302–6. 11967683 10.1007/s004640000203

[61] McMillan MT, Zureikat AH, Hogg ME, et al. A propensity score–matched analysis of robotic vs open pancreatoduodenectomy on incidence of pancreatic fistula. *JAMA Surg.* 2017;152:327–35. 28030724 10.1001/jamasurg.2016.4755 PMC5470429

[62] Lof S, Claassen L, Hannink G, et al. Learning curves of minimally invasive distal pancreatectomy in experienced pancreatic centers. *JAMA Surg*. 2023;158:927–33. 37378968 10.1001/jamasurg.2023.2279 PMC10308297

[63] Stolzenburg JU, Holze S, Neuhaus P, et al. Robotic-assisted versus laparoscopic surgery: outcomes from the first multicentre, randomised, patient-blinded controlled trial in radical prostatectomy (LAP-01). *Eur Urol*. 2021;79:750–9. 33573861 10.1016/j.eururo.2021.01.030

[64] Ahmed I, Hudson J, Innes K, et al. Effectiveness of conservative management versus laparoscopic cholecystectomy in the prevention of recurrent symptoms and complications in adults with uncomplicated symptomatic gallstone disease (C-GALL trial): pragmatic, multicentre randomised controlled trial. *BMJ*. 2023;383:e075383. 38084426 10.1136/bmj-2023-075383 PMC10698555

