# Supplementary material for: Surgical and Oncologic Outcomes of Tumescence and Sharp Dissection Versus Electrocautery Dissection in Minimal-Access Nipple-Sparing Mastectomy with Immediate Prosthesis Breast Reconstruction: A Real-World Retrospective Cohort Study
Source: Ann Surg Oncol. 2025 Jun 25;32(9):6660–70. doi: 10.1245/s10434-025-17680-4 (PMC12317917; doi:10.1245/s10434-025-17680-4)
Supplement: Supplementary file 1 — Supplementary file1 (DOCX 19 kb) [file 10434_2025_17680_MOESM1_ESM.docx]

**Supplementary Table 1. Stratified analysis for risks of necrotic complications of patients receiving minimal access nipple-sparing mastectomy with** **immediate prosthesis breast reconstruction with tumescence and sharp vs electrocautery dissection before propensity score matching**

| **Parameter** | **Odds Ratio (95% CI)** | | **P value** |
| --- | --- | --- | --- |
| Age, y |  |  | |
| ≤50 | 1 [Reference] |  | |
| 51-60 | 1.53 (1.00-2.33) | 0.049* | |
| 61-75 | 2.31 (1.16-4.60) | 0.017* | |
| BMI, kg/m^2^ |  |  | |
| ＜24 | 1 [Reference] |  | |
| 24-27.9 | 0.73 (0.46-1.16) | 0.183 | |
| ≥28 | 1.26 (0.73-2.18) | 0.403 | |
| Smoking status |  |  | |
| Never smoker | 1 [Reference] |  | |
| Former smoker | 1.46 (0.32-6.58) | 0.624 | |
| Current smoker | 3.50 (1.51-8.09) | 0.003* | |

Abbreviations: BMI, body mass index; CI, confidence interval.

**Supplementary Table 2. Odds ratios for necrotic complications among patients receiving minimal access nipple-sparing mastectomy with immediate prosthesis breast reconstruction with tumescence and sharp vs electrocautery dissection after propensity score matching**

|  | **Odds Ratios (95% CI)** | | |
| --- | --- | --- | --- |
| **Parameter** | **Crude^a^** | **Crude^b^** | **Adjusted^c^** |
| Tumescence and sharp dissection | 0.40 (0.24-0.67) | 0.39 (0.23-0.65) | 0.39 (0.23-0.66) |

a Adjusted for age.

b Adjusted for current smoker.

c Adjusted for age, BMI, current smoker, diabetes, tumor size, type of cancer, hormone receptor status, ERBB2 status, preoperative chemotherapy, postoperative chemotherapy, estimated blood loss, and duration of operation.
